# Inflammation and RONS Dysregulation by Redox Enzymes as Mechanistic Links in HIV-1–Cancer Comorbidity

**DOI:** 10.3390/pathogens15040423

**Published:** 2026-04-14

**Authors:** Charles Gotuaco Ang, Shreya Eyunni, Irwin M. Chaiken

**Affiliations:** 1 Department of Biochemistry and Molecular Biology, College of Medicine, Drexel University, Philadelphia, PA 19102, USA; se528@drexel.edu; 2 College of Arts and Sciences, Drexel University, Philadelphia, PA 19104, USA

**Keywords:** cancer, comorbidity, disulfides, HIV-1, Protein Disulfide Isomerase, redox, reactive oxygen/nitrogen species, thiols, thioredoxin

## Abstract

Antiretroviral therapy (ART) effectively controls Human Immunodeficiency Virus Type-1 (HIV-1) infection in people with HIV-1 (PWH), preventing the progression of their infections to AIDS. However, as PWH age, they experience lifestyle- and age-related diseases, notably various types of cancer beyond those traditionally associated with AIDS, with greater incidence and mortality than their non-HIV-1-positive counterparts, despite effective arrest of HIV-1 infection by ART. Dysregulation of redox enzymes presents an underexplored linkage between HIV-1 infection and cancer comorbidity, impacting reactive oxygen/nitrogen species (RONS) management, inflammation, immune function, and mitochondrial function. Chronic HIV-1 infection increases both RONS production and RONS neutralization responses, accelerating development of a sustained RONS-rich environment that still possesses sufficient dampening to prevent outright cytotoxic effects. Such an environment promotes both tumor proliferation and resistance adaptations to chemo- and radiotherapies. This review considers the effects of chronic HIV-1 infection on redox enzyme function and links these effects to tumorigenic mechanisms as potentially shared pathways. We then examine current methods of modulating redox function, consider how these could potentially impact both HIV-1 infection and cancer progression, and lastly propose future methods of co-treatment that could be explored.

## 1. Introduction: Cancer in People with HIV-1

Comorbidities are a frequent (weighted average 33.4%) complication in people with cancer, with major impacts on risk, screening, diagnosis, and treatment [1]. People with Human Immunodeficiency Virus Type 1 (HIV-1) (PWH) have increased cancer risk and poorer outcomes in several cancer types, thought to result from chronic inflammation and immunosuppression, despite effective control of HIV-1 infection progression by antiretroviral therapy (ART) [2,3,4].

In the context of PWH, types of cancer can be described as “AIDS-defining cancers” (ADCs) or “non-AIDS-defining cancers” (NADCs), where the former are mechanistically linked to immunodeficiencies engendered by HIV-1 infection, but the latter are not [5,6]. Typical ADCs include Kaposi sarcoma, aggressive B-cell non-Hodgkin lymphomas, and cervical cancer; diagnosis with an ADC is largely synonymous with progression of HIV-1 infection to AIDS [7]. NADCs include more typical age- and lifestyle-related cancers, such as cancers of the lung, breast, anus, skin, liver, and Hodgkin lymphomas [7,8]. Notably, ADC rates in PWH have fallen with the emergence and availability of effective ART, while NADC rates have continued to rise in PWH, even after extended use of ART [6,9]. Furthermore, population-based studies have demonstrated worse outcomes (increased morbidity and mortality) from NADCs in PWH than in people without HIV-1 [2,3,4,8,9,10,11]. Though the exact mechanisms driving enhanced NADC development and progression in PWH are still being explored, HIV-1-related immune activation, chronic inflammation, and oxidative stress may be important contributing factors [12,13].

**Figure 1:**
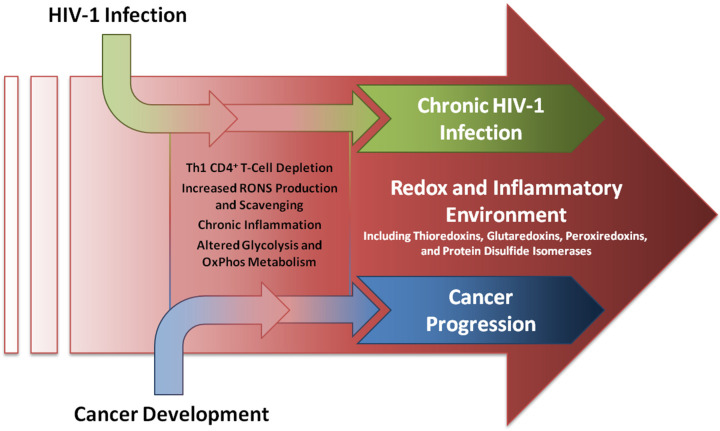
**Schematic depicting HIV-1 infection and cancer development in a shared redox and inflammatory environment.** HIV-1 infection induces a number of metabolic changes with effects on the redox and inflammatory environment. These effects parallel and overlap with metabolic changes also enacted by several types of cancer, and by contributing to a shared redox and inflammatory environment, can exacerbate the later development and progression of cancer.

The extended systems of the redox enzymes Thioredoxin (TXN, TXN1, Trx1) and Protein Disulfide Isomerase (PDI, P4HB, PDIA1–6) may serve as mechanistic links between the two diseases [14,15]. While the HIV-1 field treats TXN1 and PDIA1 similarly mechanistically insofar as their enzymatic functions (rearranging bonds on HIV-1 proteins and their host targets to facilitate infection), cancer research often categorizes them separately, with TXN1 scavenging free radicals and oxidizers [16] and PDIA1 facilitating protein folding machinery and preventing the unfolded protein response [17]. Nonetheless, the overall redox linkage between HIV-1 and cancer can be depicted as shown schematically in Figure 1, with HIV-1 infection altering the shared redox and inflammatory environment that may promote cancer development and progression.

In cancer, TXN1 functions in cell proliferation and was found to be overexpressed in aggressive tumors, and PDIs have similarly been implicated in cancer cell migration and invasion [14,18,19,20,21]. These enzymes are often upregulated to compensate for oxidative stress conditions, which are characteristic of both HIV-1 infection and tumor microenvironments [14,22,23,24,25,26,27,28]. Because TXN and PDI enzyme systems are mechanistically active in both HIV-1 pathogenesis and tumor biology, they represent promising targets for dual-action therapies aimed at treating both diseases simultaneously [14,22,29,30,31,32,33]. The study of specific molecules that can inhibit TXN and PDI enzymes in cancer and HIV-1, as well as the similarities in the inhibitors for both diseases, can help identify mechanisms for co-treatment. Though discussions regarding redox inhibition and immune system inhibition as potential options for co-treatment have been limited, ideas have been proposed [14,15,34].

## 2. The Redox Environment

### Under Normal Conditions

The body’s redox and inflammatory environment is among the factors influencing both HIV-1 infection and cancer development, and furthermore is affected by both HIV-1 infection and cancer development, promoting certain proteins and cellular responses to facilitate the progression of HIV-1 infection and cancer growth, respectively [23,26,35,36,37]. Neither of these changes occurs in a vacuum, however, and alterations to the redox environment can have far-reaching consequences on comorbidities and coinfections in the body.

The local redox environment regulates the movement of electrons in biological systems, notably in the formation and breaking of protein disulfide bonds [14,38,39,40,41,42,43]. Disulfide bonds are covalent linkages between terminal thiol (-SH) groups of cysteine residues in proteins and play diverse roles in stabilizing protein structure, regulating enzymatic activity, and enabling allosteric control of function [44,45,46,47,48]. Another function of redox enzymes is in the body’s defense against excess quantities of reactive oxygen and nitrogen species (RONS), which can cause excess oxidation [17,38,42,49,50]. RONS result from a variety of sources, including electrons leaking and forming O_2_^●−^, ^●^OH, NO_2_^●^, ONOO^−^, and other reactive species as a rare byproduct of oxidative phosphorylation, purposeful production by NADPH oxidases in response to bacterial or viral infection, nitric oxide synthases, or exposure to radiation, environmental pollution, and certain drugs, which can chemically attack and damage cellular components such as DNA, lipids, and proteins [49,50,51,52,53,54]. While limited quantities of RONS are considered normal products and byproducts in many cellular and signaling functions, redox enzymes function in metabolic systems that scavenge RONS to limit unwanted damage [55,56].

The active sites of TXN1 and PDIs contain a signature “Thioredoxin fold motif” -C-X-X-C- (often cysteine–glycine–proline–cysteine), containing a reactive dithiol from the cysteines, enabling donation of electrons to reduce other disulfide bonds while oxidizing the pair of thiols (2× -SH) into a single disulfide bond (-S-S-) [50,57,58,59]. This reduction is a key tool in the endoplasmic reticulum (ER) to ensure that synthesized proteins attain their correct native, disulfide-stabilized conformations; otherwise, misfolded proteins remain vulnerable to reduction in exposed disulfide bonds, forcing another cycle of refolding [46,47,48]. Even after exiting the ER, oxidized or reduced protein disulfide bonds may be necessary in order to maintain conformation or to modulate protein structure and function in “allosteric” disulfide bonds [44,45,60].

Thioredoxin and its related proteins are typically considered in the context of RONS management and protection [16,26,61,62]. Recent reviews on the network of thioredoxin-1, thioredoxin reductase (TXNRD1), thioredoxin interacting protein (TXNIP), peroxiredoxins (PRX), as well as glutathione (GSH), glutathione peroxidase (GPX), glutaredoxin (GRX), and glutathione reductase (GSR) in their role of RONS management include Jomova et al. [63], Li et al. [64], and Muri and Kopf [26]. To briefly describe RONS neutralization, O_2_^●−^, the most common reactive oxygen species, is converted by superoxide dismutase into peroxides (H_2_O_2_ and ROOH), which are then reduced by catalase, glutathione peroxidase, or peroxiredoxin, into H_2_O and ROH species; having reduced H_2_O_2_ into H_2_O, the oxidized GPX and PRX are regenerated into reduced GPX and PRX by using glutathione and reduced TXN as electron donors, which themselves are then re-reduced by glutathione reductase and thioredoxin reductase using NADPH as their electron donors [50,52,65].

TXN1 and GSH expression are both upregulated by Nuclear Factor Erythroid 2-Related Factor 2 (NRF2), which itself is activated during exposure to RONS, causing dissociation from Kelch-like ECH-Associated Protein 1 (KEAP1) and nuclear translocation [66]. Thioredoxin Interacting Protein (TXNIP) serves as a direct inhibitor of TXN1, but also marks NRF2 for ubiquitinylation and degradation [67].

In contrast to the RONS scavenging that GPX, PRX, GSH, and TXN are involved in, Protein Disulfide Isomerase enzymes (PDI/P4HB/PDIA1, PDIA2–) are primarily implicated in facilitating correct protein folding during protein production in the ER, although small quantities are also found in the nucleus and cytoplasm, and PDIs may be shuttled to the cell surface or extracellular environment to function as chaperones [17,23].

## 3. Redox as a Factor of HIV-1 Infection and Control

HIV-1 infection results in upregulation of oxidative stress proteins, including TXN and PDI system enzymes, which have been detected even in PWH on ART [68,69,70,71,72].

### 3.1. Redox Interaction with HIV-1 Entry Mechanisms and Relevance to ART Control

The HIV-1 lifecycle spans multiple steps, first influenced by the host’s redox environment, and later influencing the host’s redox environment [61]. On the surface of CD4^+^ T-cells, oxidoreductases such as TXN1 and PDIA1 reduce disulfide bonds within the CD4 receptor, leading to its monomerization and enabling it to interact with coreceptors on antigen-presenting cells [73,74]. However, this same disulfide reduction primes CD4 for interaction with HIV-1 Env [75]. HIV-1 Env gp120 also requires redox enzyme-catalyzed cleavage of disulfides for correct folding and function before CD4 encounter [75,76,77,78].

Upon an encounter of an HIV-1 viral particle with host target cells, CD4^+^ T-cells or macrophages, the HIV-1 Env protein interacts with CD4 and a coreceptor protein, either C-C chemokine receptor type 5 (CCR5) or C-X-C chemokine receptor type 4 (CXCR4), to induce fusogenic transformations that will allow entry of the HIV-1 genetic material into the host cell. Inhibition of redox enzymes, either TXN1 (favored in macrophages) or PDIA1 (favored in resting CD4^+^ T-cells), at this stage can inhibit the fusogenic transformation of Env, halting infection and reflecting which redox enzyme is more abundant on each cell type [32,79,80,81]. Specifically, the redox enzymes appear to target the C296/C331 disulfide pair for reduction in order to trigger rearrangement [82]. The window for this may be temporally limited, requiring conformational activation of Env by CD4/coreceptor before TXN1 or PDIA1 can attack C296/C331 [83]. A second disulfide exchange by TXN1 may also be required in the D2 domain of CD4 after HIV-1 Env binding [76].

Despite PWH being on ART control and even aviremic, soluble gp120 can still be found circulating in their blood and tissues, which can still induce production of pro-inflammatory markers on binding to host CD4, and even trigger “bystander” antibody-dependent cell cytotoxicity against cells binding the soluble gp120 [84,85,86,87]. This may result from the production of spontaneously shed soluble gp120 by defective proviruses, even in ART-controlled individuals, and may be one of the ways chronic HIV-1 infection still influences inflammation, the redox environment, and immune recovery, despite ART preventing further productive infection [85]. Entry mechanisms and inhibitors targeting the HIV-1 Env protein may therefore still have relevance to the health and treatment of PWH on ART control; however, the evaluation parameters for drugs and interventions will need to adapt beyond entry to also address the production, processing, and immunogenicity of HIV-1 Env.

### 3.2. Redox Influence on HIV-1 Transcription Mechanisms

Within the cell, TXN1 translocates to the nucleus upon cellular activation, where it is able to reduce and activate transcription factors, including the Nuclear Factor Kappa-light-chain-enhancer of activated B cells (NF-κB), which functions as a master regulator of HIV-1 transcription [61]. Furthermore, increased cell signaling and viral protein production require additional metabolic activity, generating further RONS, for which increased amounts of reduced TXN1 and GSH can compensate, masking RONS levels that would otherwise trigger apoptosis or immune clearance [14,27,88]. This additionally serves to counterbalance the effects of the HIV-1 Transactivator of Transcription (Tat) protein, which is secreted by infected cells into the circulation, where it can downregulate NRF2-controlled antioxidant genes in bystander cells, including Heme-oxygenase-1, NADPH Dehydrogenase Quinone 1, and Sulfiredoxin-1 [89]. Gp120 also enhances RONS production by upregulating the production of the enzymes proline oxidase and NADPH Oxidases 2 and 4 [35].

### 3.3. HIV-1 Infection Effects on Glycolysis/Oxidative Phosphorylation

HIV-1 is also known to alter both glycolysis and oxidative phosphorylation in infected cells, and more metabolic activity is required for virus production [35]. Glycolysis is increased by upregulation of Glucose Transporter Type 1 (GLUT1), with a possible mechanism being CXCR4 or CCR5 signaling via HIV-1 Env gp120 binding [90], as well as upregulated hexokinase activity [91]. This shift toward aerobic glycolysis also improves the packaging and budding of functional viral particles, in particular for the inclusion of HIV-1 Env and Reverse Transcriptase proteins [92].

In oxidative phosphorylation, HIV-1’s Tat protein reduces calcium signaling and phosphorylates Protein Tyrosine Phosphatase Interacting Protein 51 (PTPIP51)/Regulator of Microtubule Dynamics Protein 3 (RMDN3), both with the effect of damaging mitochondria and increasing the accumulation of RONS [35]. HIV-1 Viral Protein R (Vpr) damages mitochondrial membranes by downregulating Mitofusin-2 and Dynamin-1-like-protein, regulators of mitochondrial fusion and fission, and binding to the mitochondrial permeability transition pore [35]. Vpr additionally induces upregulation of the transcription factor HIF-1 (hypoxia inducible factor 1), which can bind and activate the HIV-1 Long Terminal Repeat, much like low hydrogen peroxide concentrations, as well as boosting mitochondrial activity and RONS production [88].

### 3.4. Redox Influence on HIV-1 Latency Control

HIV-1 latency itself (dormant HIV-1 state) is also affected by oxidative stress, as latency is often associated with the downregulation of glycolysis, lower glucose uptake and lower ATP production [61,88]. The HIV-1 protein Tat also enhances transcription and elongation, but functions more effectively in an oxidized state; TXNRD1 reduces Tat’s disulfide bonds, potentially maintaining latency [61].

High levels of RONS (requiring more peroxiredoxin and TXN to scavenge) induce TXNIP to shift from targeting TXN to targeting and binding GLUT1 for internalization and lysosomal degradation; however, the effect of TXN inhibition on latency reversal depends on cell type, where TXN inhibition in latently infected primary T-cells reinforces latency, while TXN inhibition in macrophages increases ROS sufficiently to stimulate NF-kB transcriptional activators, highlighting the importance of the baseline RONS level, antioxidant capacity, and redox response in the reservoir cells [61].

### 3.5. Th1 CD4^+^ T-Cell Depletion

Massive CD4^+^ T-cell depletion is probably the signature effect of HIV-1 infection, and despite great advances in ART development, recovery in CD4^+^ T-cell count and functionality may still not reach the level of people who are HIV-1-negative [93,94]. As measured by a recent study in Ethiopia, while being on an ART regimen greatly benefited people with HIV-1 in terms of controlling viral load and restoring CD4^+^ T-cells, their rebounded number of CD4^+^ T-cells was still significantly lower than in people without HIV-1 (CD4^+^ T-cell counts in cells/µL: HIV-1-Negative 831 ± 106; HIV-1-Positive, ART-Naïve 281 ± 127; HIV-1-Positive, On ART 442 ± 210) [95].

CD4^+^ T-cell functions vary depending on their differentiated subtype, which itself is determined by the surrounding immune environment. Th1 differentiation is primarily the subset that targets intracellular pathogens such as viruses, as well as tumors [96,97,98]. Th1-differentiated CD4^+^ T-cells are preferentially infected by HIV-1 during early asymptomatic infection due to increased CCR5 expression and dominance of M-tropic HIV-1 in early infection [99], causing an ultimate overall Th1 to Th2 “shift” as Th1 cells are depleted by HIV-1 infection responses. IFN-γ (the primary Th1-inducing cytokine) induces TXN1 production, which in turn stimulates IL-12 production from macrophages (another Th1-inducing cytokine, also induced through NF-κB signaling) [100]. TXN1 also inactivates IL-4 (the primary Th2-inducing cytokine) via a reduction in its C46-C99 disulfide [101] (C70-C123 UniProt), reducing Th2 differentiation and thereby cross-promoting Th1 differentiation, which then can be infected by HIV-1, further driving CD4^+^ T-cell depletion.

PWH show significantly elevated RONS in cellular and mitochondrial compartments of CD4^+^ T-cells, compared to healthy subjects, as well as significantly reduced expression of superoxide dismutase 1 (SOD1) and apurinic/apyrimidinic endonuclease 1 (APE1), limiting capacity for RONS neutralization [98].

### 3.6. ART Interactions

The chronic use of ART itself can also contribute to oxidative stress and mitochondrial toxicity and dysfunction [35,102]. Particularly early in the ART era, increased oxidative stress was observed in people with HIV-1 on nucleoside reverse transcriptase inhibitor (NRTI), non-nucleoside reverse transcriptase inhibitors (NNRTI), and protease inhibitor (PI)-based ART regimens [103,104,105], though in vitro experiments have shown that protease inhibitors cause large amounts of oxidative stress as well [106,107,108].

Lombardi et al. recently evaluated oxidative stress in PWH receiving modern, long-term ART, who still showed signs of elevated oxidative stress, though not without nuances [109]. Again, protease inhibitors were associated with higher oxidative stress, while integrase strand-transfer inhibitors (INSTIs) appear to cause less oxidative stress, particularly when used in dual, rather than triple, therapy formulations [109,110].

Coburn et al. recently assessed whether cancer risk in PWH was affected by types of ART (limited to protease inhibitors and non-nucleoside reverse transcriptase inhibitors), but found no significant difference in cancer risk [111]. Further studies, particularly including current first-line INSTI regimens and mechanistic linkages, are still needed.

Another study shows that newer NNRTIs may have mixed results as a drug class, as efavirenz and rilpivirine reduced insulin release and cell viability, but increased RONS generation and apoptosis in rat insulinoma pancreatic beta cells, although the NNRTI doravirine and NRTIs tenofovir disoproxil fumarate and emtricitabine had no similar effects [112]. Rilpivirine was further predicted to inhibit mitochondrial ATP synthase by in silico docking [112].

## 4. Redox as a Factor of Cancer Development and Progression

### 4.1. Graded Response to RONS: Cellular and Immune Signaling vs. Tumorigenesis vs. Cell Death

Inflammation and reactive oxygen/nitrogen species (RONS) are topics intimately connected with cancer development and progression, but the cellular response to oxidative stress varies depending on both the cell types exposed and the concentration of RONS exposure [42,63,113,114,115,116,117,118]. This includes activation of a number of diverse transcription factors, including NRF2, activator protein (AP-1), NF-κB, HIF-1, and tumor suppressor protein p53 (TP53) in roughly escalating order [63].

As discussed previously, RONS can be purposefully generated for normal immune and signaling purposes, causing a moderate, manageable increase in RONS [42,119,120]. This is the level of NRF2 activation, where the TXN and GSH systems are upregulated to support RONS neutralization [63]. Furthermore, NRF2 activation can also upregulate production of ATP-Binding Cassette (ABC) transporters, which can facilitate chemotherapy resistance by exporting drugs [42].

However, higher levels of RONS can result in oxidative damage to DNA, a key contributor to cancer development, particularly in oncogenes and tumor resistance genes, disrupting normal patterns of cellular replication and remodeling. In scavenging RONS, the TXN redox system and related redox enzymes provide protection, but also potential masking of elevated RONS levels once cancer develops [38,121,122,123,124]. RONS levels are also upregulated during apoptotic cascades in various cancer cells, and, because the cascade is initiated in the mitochondria of the cell, it is predicted that cancer’s RONS levels also originate in the mitochondria through the electron transport chain and NADPH oxidases due to increased metabolism and activity [125]. AP-1, NF-κB, and HIF-1 may also be activated at these higher levels of RONS exposure [63]. AP-1 activation can have mixed effects, including increased GSH synthesis and RONS scavenging, but also enhancing cellular proliferation and migration [63]. NF-κB transcription factors upregulate proinflammatory cytokines and chemokines and promote cellular proliferation, angiogenesis, and epithelial–mesenchymal transitions, while inhibiting apoptosis via upregulated gene products that can block cytochrome c-/apoptosis-based chemotherapeutics [63,126]. HIF-1 enhances a number of proangiogenic genes, which can assist in providing blood flow for tumor development [63].

Finally, at the highest levels of RONS accumulation, damage to DNA, lipid peroxidation, protein aggregation, and mitochondrial dysfunction can become so severe that any of several regulated cell death pathways could be activated to kill the cell, such as by activation of tumor suppressor protein p53—a strategy explored in developing pro-oxidant cancer treatments [42,63,117,118].

### 4.2. Diverse Tolerances for RONS and Redox Capacities by Cell Type

Despite this graded response to RONS being known, threshold values are harder to define. The physiological range for RONS varies widely by cell type, even by cell compartment, influenced by the capacity to neutralize RONS, including catalase, peroxidases, thioredoxin-1, glutathione, and glutathione reductase activities [120,127]. Tumor cells exhibit a higher basal level of RONS compared to healthy cells, and their rate constants for removing hydrogen peroxide (as a proxy for RONS in general) vary by cell type, but are roughly half those of their non-tumor counterparts [127,128,129].

Nor is there much of a standardized method for determining thresholds for RONS and redox capacities, particularly for distinguishing potentially tumorigenic DNA damage and regulated cell death levels of RONS exposure. Caco-2 (human colon carcinoma) cells tolerated up to 100 µM hydrogen peroxide without incurring DNA damage, as detected by comet assay [130]. Cultured MCF-7 tumor and MCF-10A normal-like human breast epithelial cell survival began dropping significantly only at 100 µM hydrogen peroxide, while in measurements of 5-hydroxymethyl-2′-deoxyuridine and single strand DNA breaks, MCF-7 tumor cells showed significant increases from 10 µM hydrogen peroxide, but MCF-10A normal-like cells did not show significant increases in 5-hydroxymethyl-2′-deoxyuridine until 200 µM, and single strand breaks did not appear until 50 µM [131]. HepG2 liver cells did not experience loss of viability (via MTT assay) until exposure to 1 mM hydrogen peroxide [132]. Additional measurements of hydrogen peroxide cell line cytotoxicities (TC_50_ values) include: SC-M1 (human stomach adenocarcinoma) 30.2 µM, HSC-3 (human oral squamous cell carcinoma) 31.6 µM, PANC-1 (human pancreatic adenocarcinoma) 60.3 µM, and Hep3B (human hepatocellular carcinoma) 48.5 µM [133].

Detection of the oxidation products nucleobase 8-oxo-7,8-dihydroguanine and nucleoside 8-oxo-7,8-dihydroguoanosine in cellular and mitochondrial DNA pools could help standardize reporting of DNA damage, using hydrogen peroxide or hydrogen peroxide equivalents as a standard measure across different cell types [134,135].

### 4.3. Targeting Redox Enzymes for Cancer Treatment

Targeting redox enzymes and their functions has been a long-standing target of interest for cancer therapy [38,42,52,113,121,123,136,137,138,139]. As discussed above, cancer cells broadly have higher basal RONS levels and lower clearance capacities than their non-cancer counterparts, resulting in a smaller pro-oxidant push being needed to obtain cancer-specific cell killing, in theory. Blood TXN1 levels have been found to be upregulated in tumor-confirmed people with cancer with non-small cell lung cancer (NSCLC) [140] and other lung [141], breast [142], liver [143], and thyroid [144] cancers. Elevated TXN1 may also indicate prostate cancer stem cells and predict disease outcome [145]. TXNRD1 has additionally been considered as a marker for breast and gynecologic cancers [146,147]. Furthermore, elevated TXN1 expression in NSCLC has been associated with “cold” tumor formation and immunotherapy resistance [25], and TXNRD1 has been measured as a strong predictor of tumor recurrence for NSCLC [148]. Lower TXNIP expression has also been associated with poorer prognosis of renal carcinoma [149], and as TXNIP is a direct inhibitor of TXN1, this would functionally elevate TXN1.

Jovanović et al. provide a review of the relatively limited clinical trials targeting TXN1 (with PX-12) and TXNRD1 (with Auranofin or Ethaselen) (see Table 1) in people with cancer [38]. PX-12 was found to be generally well-tolerated, but the best results were stable disease in two people with cancer (of 17), and required an elevated level of plasma TXN1 prior to treatment for those results [150,151,152,153,154,155]. Subsequent studies were terminated due to the difficulty of continuous IV infusion and predicted severe side effects in longer term administration. Auranofin has had a well-established safety profile since its FDA approval for rheumatoid arthritis in 1985, and was studied in combination with the mTor inhibitor sirolimus (rapamycin) against small and non-small cell lung cancers [156] and ovarian cancer [157], though, as recently reported, the ovarian cancer study was terminated early after showing no tumor response to the combination treatment [158]. Ethaselen is another TXNRD1-targeting inhibitor, primarily studied in China, and while a study has been listed as completed on ClinicalTrials.gov as of 2022, no data or results have been published as yet [159,160,161].

Arsenic trioxide is another drug inhibiting TXNRD1 (see Table 1) and enhancing RONS as a potential anti-cancer mechanism, with a history of clinical use against acute promyelocytic leukemia, and is being investigated for use against other cancers [162,163,164]. Against HIV-1, arsenic trioxide has been proposed for clinical trial testing as a latency reversal agent due to its proinflammatory effects, but no updates have been provided since 2022 [165].

Overall, of the published trials, the findings of the PX-12 studies demonstrated that there was a benefit (disease stabilization) in targeting TXN1, but study stratification to pre-identify people with cancer who have elevated TXN1 would improve the approach and likelihood of tumor response [150,151,152,153,154,155].

**Table 1:** Redox-Targeting Drugs in Cancer and HIV-1 Clinical Trials.

Drug	Target	Cancer Trials	HIV-1 Trials
PX-12	TXN1	Advanced Solid Tumors [151,153,154] Gastrointestinal [152] Pancreatic Adenocarcinoma [150,155]	-
Arsenic Trioxide	TXNRD1	Acute Myeloid Leukemia [166] Acute Promyelocytic Leukemia [167,168,169,170,171,172,173,174] Glioma [175] Neuroblastoma [176] Ovarian [177] Pancreatic Adenocarcinoma [178] Pediatric Cancers [179]	Latency Reversal [165]
Auranofin	TXNRD1	Glioblastoma [180] Leukemia [181] Ovarian [157,158,182] Small and Non-Small Cell Lung Cancer [156]	HIV-1 Cure [183,184]
Ethaselen	TXNRD1	Non-Small Cell Lung Cancer [159]	-

### 4.4. Anti-Tumor Roles of CD4^+^ T-Cells

CD4^+^ T-cells play a central role in both the coordination of immune responses and the surveillance of metastatic tumors. Hence, HIV-1-induced depletion of CD4^+^ T-cells will directly impact cancer development and progression [185]. CD4^+^ T-cells function in antitumor immunity by killing tumor cells and reducing tumor angiogenesis [186]. Though CD8^+^ T-cells are the primary effector cells for the adaptive immune response against tumors [186], CD4^+^ T-cells have recently been recognized as contributing to antitumor cell killing within the tumor microenvironment (TME) [187]. CD4^+^ T-cells that express cytotoxic molecules are called CD4^+^ cytotoxic lymphocytes (CD4^+^ CTLs), and are thought to originate from Th1 CD4^+^ T-cells or naïve CD4^+^ T-cells that are treated with IL-2 (Th0 conditions) in vitro, based on chronic viral infection studies [187]. CD4^+^ CTLs express Th1 cytokine-related genes and secrete interferon-gamma (IFN-γ) and interleukin-12 (IL-12), both of which increase cytotoxic functionality. This allows CD4^+^ CTLs to either kill tumor cells directly by activating metabolic pathways, such as tumor necrosis factor (TNF)-related apoptosis-inducing ligand (TRAIL), or kill tumors indirectly by recruiting immune cells to the TME and enhancing antitumor immune responses through IFN-γ production. CD4^+^ T-cells can also exert their antitumor effects through tumor blood vessel constriction, the inhibition of angiogenesis, and the activation of tumor cell senescence. The capacity of HIV-1 to kill CD4^+^ T-cells is thus a major factor in the rise in cancer occurrence in PWH.

### 4.5. Other Inflammatory Comorbidities

Mechanistically, comorbidities may contribute to cancer development by influencing inflammation and RONS management. This can occur not only for chronic HIV-1 infection, but also type 2 diabetes and chronic obstructive pulmonary disease (COPD) [27,188,189,190]. Chronic inflammation and insulin resistance in type 2 diabetes are thought to contribute to additional cancer risk and mortality [191,192]. COPD causes both lung and systemic inflammation, with elevated antioxidant capability (including the TXN system) as a compensatory mechanism [190]. Chronic HIV-1 infection, type 2 diabetes, and COPD all elevate TXN1 and/or TXNRD1 expression [70,190,193], which may also be secreted into the plasma to affect additional cells.

## 5. Current and Future Research Targeting Redox Enzymes in Co-Infection

While the repurposing of drugs has been a favored approach to incorporating redox enzyme targeting into cancer and HIV-1 treatments, these are usually done with respect to just one of the two diseases [180,194,195,196,197,198,199]. However, with the increase in cancer development in PWH on ART, a combinatorial approach to therapies could advance science and treatment possibilities. Additional attention to PWH’s oxidative stress, both in further investigations of ART mechanisms impacting RONS and redox enzymes, as well as the potential use of redox enzyme-targeting drugs, could greatly manage cancer risk. This need is only further enhanced when PWH actually develop cancer.

### 5.1. Targets and Translation

Analyses of existing people with HIV-1 and cancer comorbidities suggest the liver, pancreas, and lung as having notably elevated cancer-specific mortality [4], and these cancers are also associated with upregulated redox enzyme profiles [200], suggesting these as ideal initial targets to establish intervention methods and systems, before broadening to additional cancer types with HIV-1 and non-HIV-1 inflammatory comorbidities. Furthermore, this would be in line with the findings of the PX-12 clinical trials in order to maximize the effect and pre-identify people with HIV-1 and cancer with elevated pre-treatment levels of redox enzymes.

### 5.2. As Supplement to Existing Therapy

Characteristics of the TME could be used to apply targeted redox enhancement or depletion, whether as a complement to conventional therapies (i.e., overcoming resistance to chemotherapy or radiotherapy) or as a treatment in itself to enhance native immune functions and to impair tumor metabolism. Supplemental to this would be an evaluation of how existing therapies and drugs can affect redox balance both systemically and in the tumor microenvironment, as this may or may not have been an initial consideration in their use.

Recently, the NRTI Efavirenz has been studied in several cancer types, including breast [201], Kaposi’s Sarcoma [202], Non-Hodgkins Lymphoma [203], pancreatic [204], and prostate [205,206]. Efavirenz functions by arresting the cell cycle and causing DNA damage due to RONS production and oxidative stress, particularly in cancer stem cells [194,207,208], and may also be able to synergize with radiotherapy [209], although this has not yet been tested in clinical trials. In contrast, development of a number of other drugs targeting RONS mechanisms in cancer for radio- or chemotherapy sensitization has since been discontinued, due to lack of clinical advantage in clinical trials, as reviewed by Kirkpatrick et al., including motexafin gadolinium, buthionine sulfoximine, Telcyta, Telintra, Disulfiram, and NOV-002 [139]

### 5.3. Translational Challenges

Redox systems are a complex and far-reaching modulator for numerous biological processes, affecting and being affected by infection, inflammation, and tumor development. Therefore, any kind of intervention, whether enhancing or inhibiting, will benefit from localized, precision targeting. Progress and advances in understanding the contents and functions of the tumor microenvironment (TME) can support this precision targeting in order to affect tumors and minimize systemic and off-target effects. Similarly, identification of HIV-1-infected cells, and critically, latently infected cells, is needed to precisely target and effect the desired redox alterations. As discussed with HIV-1 latency, both reactivation and latency enhancement are possible with redox signaling, but depend on both targeting the correct cell reservoir (macrophages or memory CD4^+^ T cells) with designed drug carriers or delivery systems and delivering the proper dosage to elicit the desired redox adjustment.

However, this is complicated by the lack of standardized measurements for oxidative DNA damage and cytotoxicity thresholds in the variety of cancerous and non-cancerous cells that may be targets for treatment. Standardized measurements of these two thresholds across a spectrum of different cell types, cancerous and non-cancerous, infected with HIV-1 (or other cancer-associated pathogens) or not, could provide useful resources for the development of redox modulation as a therapeutic approach and facilitate translation of therapy development between different cancer specialties.

## Data Availability

No new data were created or analyzed in this study.
